# An empirical investigation into the role of subjective prior probability in searching for potentially missing items

**DOI:** 10.1098/rsos.150100

**Published:** 2015-07-22

**Authors:** T. R. Fanshawe

**Affiliations:** Nuffield Department of Primary Care Health Sciences, University of Oxford, Oxford, UK

**Keywords:** subjective probability, prior probability, prevalence, visual search, decision error

## Abstract

There are many examples from the scientific literature of visual search tasks in which the length, scope and success rate of the search have been shown to vary according to the searcher's expectations of whether the search target is likely to be present. This phenomenon has major practical implications, for instance in cancer screening, when the prevalence of the condition is low and the consequences of a missed disease diagnosis are severe. We consider this problem from an empirical Bayesian perspective to explain how the effect of a low prior probability, subjectively assessed by the searcher, might impact on the extent of the search. We show how the searcher's posterior probability that the target is present depends on the prior probability and the proportion of possible target locations already searched, and also consider the implications of imperfect search, when the probability of false-positive and false-negative decisions is non-zero. The theoretical results are applied to two studies of radiologists' visual assessment of pulmonary lesions on chest radiographs. Further application areas in diagnostic medicine and airport security are also discussed.

## 1. Introduction

Visual search is a common task both in everyday life and in the scientific research environment [1,2]. A particularly common scenario is one in which the search ‘target’ may or may not be present. This arises in applications ranging from quality control in engineering [3] and baggage scanning in airport security [4] to the examination of medical images for broken bones [5] and cancerous lesions [6]. In each case, there is a trade-off between the accuracy of the search and the time taken to complete the search task.

The question to be addressed in this paper is as follows: how long should a searcher continue to search, given that the target has not yet been found? At what point should the search be terminated and the target declared to be absent? Although it might appear desirable to conduct a thorough search of *all* possible locations, enabling the searcher to determine with certainty whether the target is present, in certain scenarios a search of this kind may be too time-consuming or too expensive. This is a particular concern if there are many such search tasks to be completed in succession, as might be the case in a busy airport or in a disease screening programme [7]. Additionally, the search strategy may be inefficient [8], leaving possible target locations unexplored, or the decision as to what constitutes a target might be subject to error [9].

It appears reasonable to think that the time spent searching might depend on the individual's level of anticipation that the target is present, which can be regarded as a prior probability. It appears natural to interpret this as a subjective Bayesian probability [10], which is gradually modified as the searcher assimilates information about the presence or absence of the target during the search.

This paper assesses how this probability might change according to the length of the search, and consequently indicate when the searcher might terminate the search entirely, as might be the case if the posterior probability falls below a suitably small value. The topic is introduced in §2 by means of a simple example based on just two possible locations, which is then extended in §3 to the case of multiple locations. Section 4 discusses the case in which the reader's ability to identify of the target is subject to error. Section 5 evaluates the approach in an analysis of data from two published studies of the assessment of pulmonary lesions on chest radiographs, and §6 is a concluding discussion of further implications for applied research.

## 2. A simple example

Suppose initially that there are only two possible locations (‘cell 1’ and ‘cell 2’) in which the object of interest (‘target’) may be hidden. Let *Z* denote the event that ‘the target is present in either of the cells’, and *Z*^c^ its converse (‘the target is absent’). Let *X*_*i*_ denote the event that ‘the target is in cell *i*’ (*i*=1,2), and $X_{i}^{c}$ its converse.

We assume that either exactly one of the cells contains the target, which occurs with prior probability *π*=P(*Z*)∈(0,1), or neither of the cells contains the target, with prior probability 1−*π*. We also assume that the cells are superficially identical, so that if the target is present, it is equally likely to lie in either of the cells (i.e. $\text{P}(X_{1}|Z)=\text{P}(X_{2}|Z)=\frac{1}{2}$).

Initially, we assume that the searcher operates without error: searching a particular cell establishes with certainty whether that cell contains the target. In §4, we will investigate the implications of relaxing this assumption. Clearly, if cell 1 contains the target, we have P(*Z*|*X*_1_)=1. Suppose that cell 1 has been searched and does not contain the target.

Application of Bayes's theorem gives

$$\begin{array}{rl} \text{P}(Z|X_{1}^{c}) & =\frac{\text{P}(X_{1}^{c}|Z)\text{P}(Z)}{\text{P}(X_{1}^{c})}\\ & =\frac{\text{P}(X_{1}^{c}|Z)\text{P}(Z)}{\text{P}(X_{1}^{c}|Z)\text{P}(Z)+\text{P}(X_{1}^{c}|Z^{c})\text{P}(Z^{c})}\\ & =\frac{(1/2)\pi }{\left(1/2)\pi +1(1-\pi \right)}\\ & =\frac{\pi }{2-\pi }. \end{array}$$

Thus, for example, if the searcher's prior probability that the target was present in one of the two cells is $\pi =\frac{1}{2}$, the posterior probability $\text{P}(Z|X_{1}^{c})$ of this event upon finding that cell 1 were empty would drop to $\frac{1}{3}$.

For an intuitive explanation of this result, we might consider the outcome of a series of ‘trials’ in each of which the target is present with probability *π*=0.5. In the long run, the target would be in cell 1 in one in every four trials; in the other three, two would be scenarios in which both cells were empty, and in the other, cell 2 would contain the target. If the first cell is empty, the first of these cannot occur, and the other three will occur with equal probability, giving the ‘answer’ of $\frac{1}{3}$. The logic shares some similarities with certain explanations of the ‘Monty Hall problem’ [11].

## 3. Extension to the case of multiple cells

Consider now the case in which there are *n* cells, of which *m* have been searched without the target being found, i.e. ${\text{X}}_{m}^{c}=(X_{1}^{c},\ldots ,X_{m}^{c})$ has been observed. The posterior probability of the target being present is

3.1 $$\begin{array}{rl} \text{P}(Z|{\text{X}}_{m}^{c}) & =\frac{\text{P}({\text{X}}_{m}^{c}|Z)\text{P}(Z)}{\text{P}({\text{X}}_{m}^{c})}\\ & =\frac{\text{P}({\text{X}}_{m}^{c}|Z)\text{P}(Z)}{\text{P}({\text{X}}_{m}^{c}|Z)\text{P}(Z)+\text{P}({\text{X}}_{m}^{c}|Z^{c})\text{P}(Z^{c})}\\ & =\frac{(n-m)/n\pi }{\left(n-m)/n\pi +1(1-\pi \right)}\\ & =\frac{(n-m)\pi }{n-m\pi }\\ & =\frac{(1-p)\pi }{1-p\pi }, \end{array}$$

where *p*=*m*/*n* is the proportion of cells that have been searched. The same formula is applicable if we envision the search area as spatially continuous rather than consisting of a set of discrete locations, provided that any information gleaned from searching target-free regions is independent of whether the target is present and where it is located.

**Figure 1.**
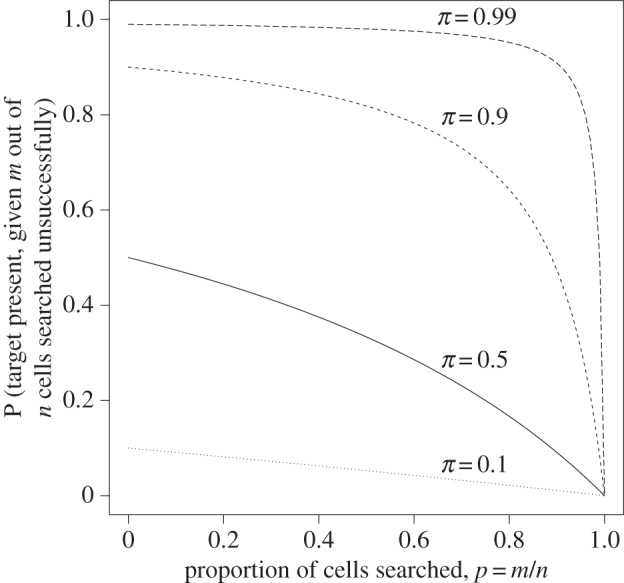
The relationship between the proportion of locations searched (*p*=*m*/*n*) and the posterior probability that the target is present, given that *p*=*m*/*n* cells have been searched without finding the target, assuming no error in detection. The lines show how the posterior probability changes for four different values of the prior probability *π*: dotted line, *π*=0.1; solid line, *π*=0.5; short dashed line, *π*=0.9; long dashed line, *π*=0.99.

Figure 1 shows how equation (3.1) varies with *p* for fixed values of *π* (R code used to create the figures is available as the electronic supporting information, where the effect of changing parameter values can be seen [12]).

If the prior probability *π* is high, the posterior probability will remain high except when an extremely thorough search is made. If *π* is large (0.99, say), searching a proportion *p*=*π* of possible locations gives a posterior probability of $p/(1+p)\approx \frac{1}{2}$, so even a fruitless search of 99% of all possible locations leaves no less than an even chance that the target will be found in the remaining 1% of locations.

Keeping *π* fixed, for small values of *p*, the function *f*(*p*)=(1−*p*)*π*(1−*pπ*)^−1^ is approximately quadratic: *f*(*p*)≈1−2(*pπ*)^2^ by Taylor expansion. The second derivative of *f* with respect to *p* is

$$\begin{array}{rl} f^{\prime \prime }(p) & =-\frac{2p\pi^{2}(1-\pi )}{{\left(1-p\pi \right)}^{3}}\\ & \leq 0\;\forall \;p \end{array}$$

and so *f* is a concave function: for a given proportion of possible locations, the more has already been searched, the greater the reduction of the posterior probability by a subsequent search. As *f*′(*p*) is a monotonically decreasing function of *p* for given *π*, the closer the searcher comes to a full search (i.e. *p*=1), the sharper the gain, particularly when *π* is also close to 1.

The probability (3.1) drops below a fixed threshold *t*<*π* when

$$p>\frac{\pi -t}{\pi (1-t)}.$$

Using *t*=0.05 for exposition (although in many scenarios a much smaller *t* may be more appropriate), this requires 52.6%, 94.7%, 99.4% and 99.9% of all possible locations to be searched if *π*=0.1, 0.5, 0.9 and 0.99, respectively.

Similar results apply if, instead of regarding each cell as equally likely to contain the target, probabilities are allowed to vary between cells, with cells searched in decreasing order of probability. The prior probabilities might be represented as a non-increasing function *π*(*x*), where *x*∈[0,1] and $\pi =\int_{0}^{1}\pi (x)\;dx$. A suitably scaled beta distribution might serve as a convenient parametrization. In this case, the results given above still apply if *p* is interpreted as the ‘*π*(*x*)-weighted’ proportion of locations searched. The proportion *p* would correspond to a search of the first 100*z*% of locations, where *z* satisfies $p=\int_{0}^{z}\pi (x)\;dx$.

## 4. Extension in the presence of decision error

In practice, a search can rarely be carried out without the possibility of errors arising. For example, the assessment of medical imaging is generally subject to both recognition errors (in which the eye fixates on the target but the searcher fails to realize that an identification needs to be made) and decision errors (in which the searcher realizes that the object under scrutiny may be the target, but incorrectly identifies its nature) [6]. Visual search error, when the searcher fails to make a visual assessment of some parts of the region, is also possible and would correspond to a reduction in the proportion *p* of possible target locations searched. Such errors may occur through human error (e.g. fatigue or lack of sufficient expertise) or through factors that are generally beyond the searcher's control (e.g. if the quality of the image as a whole is poor, or the target itself is to some extent hidden or distorted). In this section, we extend previous results to allow for both ‘false positive’ and ‘false negative’ errors: situations in which, respectively, the searcher decides a target is present when it is not, and the searcher fails to identify a target that is present.

*X*_*i*_ still denotes the event that ‘the target is in cell *i*’, but now this is not directly observable. Instead, either *Y*_*i*_, the event that the searcher decides that cell *i* contains the target, or $Y_{i}^{c}$, its converse, occurs. Let $q=\text{P}(Y_{i}|X_{i}^{c})$ be the probability of a false positive decision, and $r=\text{P}(Y_{i}^{c}|X_{i})$ be the probability of a false negative decision. The searcher's decisions in different cells are assumed to be independent, and *q* and *r* are constrained to satisfy |*q*−*r*|<1, else all decisions would be either always positive or always negative.

Corresponding algebraic expressions to those in §§2 and 3 can be derived. Appendix A contains details of the derivation. In the case of only two cells, the posterior probabilities in the case that cell 1 is empty are

4.1 $$\text{P}(Z|Y_{1}^{c})=\frac{(1-q+r)\pi }{2(1-q)-(1-q-r)\pi }$$

and

4.2 $$\text{P}(Z^{c}|Y_{1}^{c})=\frac{2(1-q)(1-\pi )}{2(1-q)-(1-q-r)\pi }.$$

Note that because of decision error, the posterior probabilities given *Y*_1_ are now non-trivial expressions:

4.3 $$\text{P}(Z|Y_{1})=\frac{(1+q-r)\pi }{2q+(1-q-r)\pi }$$

and

4.4 $$\text{P}(Z^{c}|Y_{1})=\frac{2q(1-\pi )}{2q+(1-q-r)\pi },$$

which are the same as equations (4.1) and (4.2) after replacing *q* and *r* with 1−*q* and 1−*r*, respectively.

As in §3, we can extend these formulae to the case in which a proportion *p* of the cells have been observed to be empty (i.e. we observe ${\text{Y}}_{m}^{c}=(Y_{1}^{c},\ldots ,Y_{m}^{c}$)):

4.5 $$\text{P}(Z|{\text{Y}}_{m}^{c})=\frac{[(1-q)(1-p)+pr]\pi }{1-q-(1-q-r)p\pi }$$

and

4.6 $$\text{P}(Z^{c}|{\text{Y}}_{m}^{c})=\frac{\left(1-q)(1-\pi \right)}{1-q-(1-q-r)p\pi }.$$

The correspondence between equations (4.5) and (4.6) and the special cases equations (4.1) and (4.2) in which $p=\frac{1}{2}$ is apparent. Even though these expressions are derived by regarding the search region as consisting of a composition of disjoint sub-regions, they do not depend on either *m* or *n*, and so retain their interpretation when the search region is spatially continuous. This is potentially important, as in applications such as medical imaging, a single global decision or diagnosis may be made in preference to a series of decisions based on subregions.

**Figure 2.**
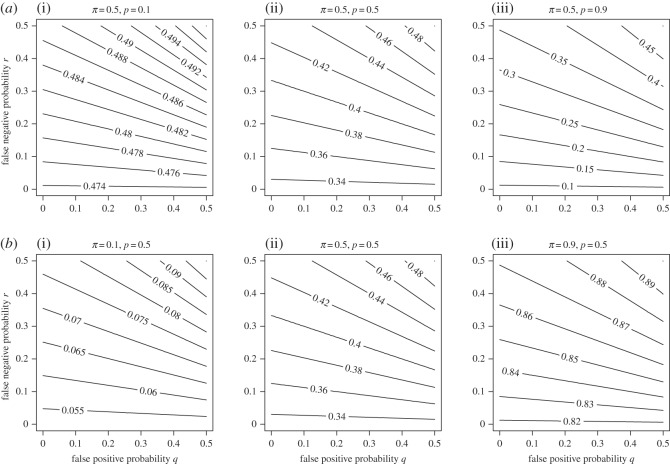
Relationship between false positive probability, false negative probability, proportion of locations searched and posterior probability. The posterior probability shows (shown by contour lines) that the target is present, given that it has not been found after searching a proportion *p* of possible locations, is shown as a function of the overall false positive probability *q*, and the false negative probability *r*. In (*a*), *p* is varied from 0.1 (i) to 0.5 (ii) to 0.9 (iii) for fixed prior probability *π*=0.5. In (*b*), *π* is varied from 0.1 (i) to 0.5 (ii) to 0.9 (iii) for fixed *p*=0.5.

**Figure 3.**
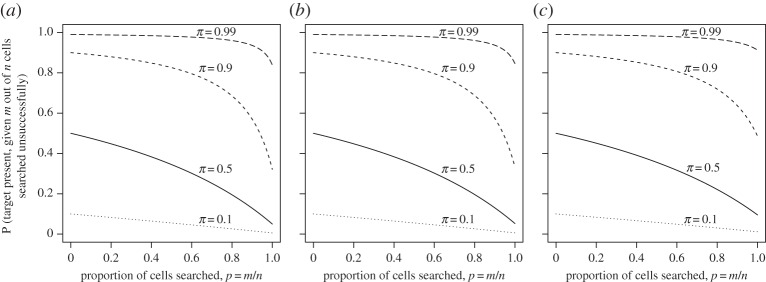
Effect of the proportion *p* of possible locations searched on the posterior probability of the target being present in the presence of decision error. Values of the prior probability *π*: dotted line, *π*=0.1; solid line, *π*=0.5; short dashed line, *π*=0.9; long dashed line, *π*=0.99. (*a*) *q*=0.05, *r*=0.05; (*b*) *q*=0.1, *r*=0.05; (*c*) *q*=0.05, *r*=0.1.

Figures 2 and 3 show how the posterior probability varies with respect to *p*, *q*, *r* and *π*. The impact of decision errors clearly increases the longer the search continues (i.e. as *p* increases), both because a larger number of assessments provides a greater chance of a false positive decision occurring and, more importantly, because if the target is present, its location is more likely to have been assessed if *p* is large. The lower panel of figure 2 shows that changing *q* and *r* has a larger effect on the posterior probability when *π* is nearer 0.5 than either of the extremes, which is intuitively reasonable: with equivocal prior opinion, the searcher's decision depends primarily on the observed data.

Figure 3 shows, for fixed, non-zero *q* and *r*, how the searcher's posterior probability changes as the search develops. It can be compared to figure 1, which represents the case in which *p*=*q*=0. The dependence on *r* can be seen particularly at high prevalence levels when the proportion searched is also high. In general, the posterior probabilities (4.5) and (4.6) depend on both the false positive and false negative error rates, even though these expressions are probabilities conditional only on a set of observations in which the target has *not* been found.

The overall pattern of results from figures 2 and 3 demonstrates that *π* has a much larger effect than both *q* and *r* at almost all levels of *p*, especially when *q* and *r* are small, as would be the case in most applications. One important exception presents itself: when a complete search has been made. In this case, in the absence of decision error, the prior probability is irrelevant, as the posterior probability is either one or zero, depending on whether the target has been found. However, figure 3 shows that this does not apply when decision error is present. The figure is plotted on the domain [0,1], with the posterior probability at *p*=1 given by

$$\text{P}(Z|{\text{Y}}_{m}^{c})=\frac{r\pi }{1-q-(1-q-r)\pi }.$$

The *prπ* term in the numerator of equation (4.5) is critical: if *π*=0.99, *p*=1 and *q*=0, increasing the false negative probability *r* from 0 to just 0.01 increases the posterior probability that the target is present from 0 to 0.5. The scenario *p*=1 has been widely studied in the diagnostic accuracy literature [13].

## 5. Application

We apply the results above to two studies by Reed *et al.* that examined the effect of prevalence expectations on the performance of radiologists' visual assessment of pulmonary lesions on chest radiographs [5,14]. The designs of the studies were similar: both required 22 experienced radiologist readers, with a minimum of 6 years' post-registration experience, to assess a sample of 30 radiographs, 15 of which contained abnormalities and 15 of which were ‘normal’. In most scenarios, prevalence information was provided to readers in advance (either $\frac{9}{30},\frac{15}{30},\frac{22}{30}$, corresponding to prevalences of 30%, 50% and 73%, respectively); there was also one scenario in which the prevalence was not revealed in advance. One of several outcome measures collected was the total duration spent scrutinizing each image.

Here, we use the total duration data for the scenarios in which numerical prevalence information was provided. We use data only from the search of the ‘normal’ images, as in most cases search of abnormal images was interrupted by the finding of at least one lesion. Therefore, we consider only cases when, unknown to the reader, no target was present. Both studies found statistically significantly increased scrutiny times at higher levels of prevalence, with readers taking an average of 8–10 s per image at the lowest prevalence level to 17–18 s per image at the highest (table 1, calculated from results given in the two papers). The results we have derived in this paper can be used to provide additional insight into the findings of the two studies of Reed *et al*. In particular, we use the average search duration data to estimate the proportion of the radiograph that is consistent with adequate search by readers at the different prevalence levels.

**Table 1.** Results and estimated proportions of locations searched, using data from the two studies of Reed *et al.*

	Reed *et al.* [14]		Reed *et al.* [5]	
prevalence, *π*_*i*_ (%)	time, *t*_*i*_ (s)	$\hat{p_{i}}$	time, *t*_*i*_ (s)	$\hat{p_{i}}$
30	10.3	0.56	8.5	0.45
50	14.9	0.81	14.5	0.77
73	17.1	0.93	18.3	0.97

**Figure 4.**
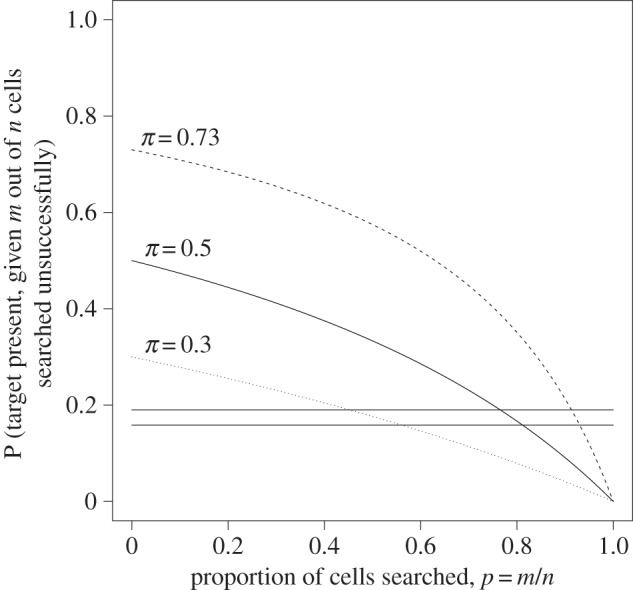
Illustration using the results of Reed *et al*. The three curves represent the relationship between proportion searched *p* and posterior probability of the target being present (dashed line: *π*=73%; solid line: *π*=50%; dotted line: *π*=30%). The points where the horizontal lines intersect the curves correspond to the proportions searched that are consistent with the mean search times at each prevalence level from the two studies of Reed *et al.* [5,14].

For clarity of exposition, we consider the results in §3 (no decision error), although the method is readily extended to the more general case in §4. Three major assumptions are required. Firstly, readers are assumed to be ‘Bayesian readers’ in the sense that, given prevalence information, their behaviour is assumed to follow the principles already described in this paper. The decision as to when to cease searching is based on the stated prevalence allied to information already accumulated during the search, as represented by the likelihood, resulting in the posterior probabilities shown in figure 4. Secondly, readers are assumed to finish searching when their posterior probability of a lesion being present reaches a particular (unknown) level *π*^⋆^, which is the same regardless of the experimental condition. Thirdly, the rate at which information is accumulated during the search is assumed to be constant over time, so that, in two separate searches, the ratio of the two scrutiny times is equal to the ratio of the proportions of possible locations searched.

Let, *i*=1,2,3 index the three prevalence levels *π*_*i*_ (30%, 50% and 73%, respectively). Let *p*_*i*_ be the proportion of possible target locations searched when the search ends, and *t*_*i*_ the time in seconds at which this occurs. The assumptions imply that, for each *i*≠*j*,

5.1 $$\frac{p_{i}}{p_{j}}=\frac{t_{i}}{t_{j}}$$

and, using (3.1), for each *i*≠*j*,

5.2 $$\frac{(1-p_{i})\pi_{i}}{1-p_{i}\pi_{i}}=\frac{(1-p_{j})\pi_{j}}{1-p_{j}\pi_{j}}:=\pi^{⋆}.$$

Solving these expressions for the *p*_*i*_ gives the estimate

$${\hat{p}}_{2}=\frac{\pi_{1}-\pi_{2}}{(t_{1}/t_{2})\pi_{1}+(1-t_{1}/t_{2})\pi_{1}\pi_{2}-\pi_{2}},$$

with estimates ${\hat{p}}_{1}$ and ${\hat{p}}_{3}$ following immediately from equation (5.1). The estimated values of these probabilities are also given in table 1. In both studies, the extent of search is consistent with the readers' posterior probability dropping to just below 20%, which corresponds to adequate search of almost all possible locations in the case *π*_*i*_=73%, but only around half of the locations in the case *π*_*i*_=30% (figure 4).

## 6. Discussion

This article provides a theoretical basis for understanding why duration of search depends on the searcher's initial expectation that the target of the search is present. That the posterior probability, when viewed as a function of the proportion searched *p* for fixed prior probability *π*, is concave is revealing, and might be seen as a ‘law of increasing returns’: the more of the region has already been searched, the greater the return, in terms of change in posterior probability, in searching a fixed proportion extra.

In treating the assessment of different cells as independent, we have not considered issues such as peripheral vision; nor have we addressed the scenario in which decision error may vary according to location, and may also be associated with the location-varying prior probability that the target is present. To achieve the latter would be algebraically cumbersome but no more difficult in principle than the scenario we have considered. We have concentrated on probabilities conditional on the event that, in the opinion of the searcher, no target has been found, and not investigated in detail the consequences of a ‘false positive’ assessment. This is primarily because in the examples that motivate this work, a ‘positive’ decision would typically lead to swift further action being taken (such as conclusive investigation of a suspect package during baggage screening) that radically alters the nature of the search task. Nevertheless, the extremely low prevalence of suspicious packages is sufficient to greatly reduce the probability of detection for imperfect searches.

There is evidence that performance can be improved by deliberately adding artificial targets to increase ‘vigilance’ [15]: inducing the observers to use a higher prior probability *π* as a means to increase *p*, in the terminology of the current paper. The value of doing so will depend on the trade-off between the respective costs of false positive and false negative decisions in any application, and is perhaps best suited to those in which false negatives, or ‘misses’, are costly errors.

There is also substantial medical literature to suggest that, in certain circumstances, so-called ‘prevalence expectations’ may affect diagnostic accuracy, and thus either the duration and thoroughness of the visual search or the way in which the findings of the search are interpreted [5,7,9]. When the prevalence of events is at an extreme, either high or low, the risk is that the searcher may be so influenced by prior expectations that scant attention is paid at all to the results of the search itself. Researchers should therefore also be aware that predictions of rare events may be influenced in the same way, perhaps making them unhelpfully conservative [16].

The application of our theoretical results to the findings in the two papers by Reed *et al.* has some limitations. We assume that the outcomes of different reads are independent, so readers' decisions are not influenced by their assessment of the cases already seen allied to the expected prevalence level. Framing prevalence information in terms of ‘expected’ or ‘population’ prevalence, rather than ‘sample’ prevalence, should help to prevent this. Other limitations relate to the three stated assumptions in §5 that underlie the analysis. In particular, although the model fits the experimental findings well, there is no intrinsic evidence that the readers, even subconsciously, follow Bayesian reasoning in making their decisions. Our results can show no more than that their search times are consistent with such reasoning. The novelty lies in providing an estimate of the proportion of locations searched corresponding to such a model, given the assumptions, which might be tested experimentally in future research and used as a means of highlighting possible reasons behind inadequate search when prevalence expectations are low.

In screening programmes, the prevalence and incidence of disease is typically very low. For example, in the National Lung Screening Trial, the annual incidence of lung cancer diagnosed by low-dose computed tomography was 0.6% [17], while a recent pilot trial in the UK assumed a 5 year incidence of 5% in a high-risk population [18]. The results in §§3 and 4 apply to assessments of individuals. Screening programmes generally assess many thousands of individuals, which in conjunction with the low prevalence of disease has the effect of producing an extremely high false positive rate, a result that has been discussed in detail elsewhere [19,20]. This well-established finding is consistent with results in §4, which show that a small false positive rate can have a substantial effect on the posterior probability, even when the diagnosis is negative.

The relationship between duration of search and diagnostic accuracy in the context is less clear, and blinded studies in this area are difficult to conduct without changing the nature of the search task [7]. There is some evidence that duration of search is associated with greater diagnostic accuracy when conducting clinical examinations for breast cancer, with a guideline of 3 min per breast recommended in order to achieve complete search, but the relationship between the duration of search and the extent of the search (in terms of possible target locations searched) remains unclear [21]. The two studies of Reed *et al.* considered in §5 used high disease prevalences, and so these results cannot be extrapolated to the lower prevalence levels that would be observed in the context of screening.

A natural extension of this work would be to link the Bayesian model for the decision-making process, proposed here, to the rich class of optimal observer models that relate to the way in which the visual search is conducted [22,23]. The results could also be extended to consider the case of multiple targets [24] or by providing information about the proportion of items searched to the searcher explicitly. These are all possible topics for future research.

## Supplementary Material

R code. File containing R code used in statistical analysis

## Data accessibility

R code used to create the figures in this paper is available as an electronic supplementary material.

## Appendix A

Here, we give a derivation of equations (4.5) and (4.6). Results (4.1) and (4.2) are special cases in which $p=\frac{1}{2}$ and so follow immediately. The proofs of equations (4.3) and (4.4) are similar.

To prove equation (4.5), we condition on the events *X*_*i*_ and $X_{i}^{c}$ that represent whether the target truly lies in cell *i*. Let ${\text{X}}_{m}^{c}=(X_{1}^{c},\ldots ,X_{m}^{c})$, the event that none of cells 1 to *m* contains the target and let ${\text{X}}_{m}^{-c}=(X_{2}^{c},\ldots ,X_{m}^{c})$, the event that none of cells 2 to *m* contains the target.

Note that the target may be contained in at most one cell. Using the Law of Total Probability and the interchangeableness of the *m* cells, we have

A 1 $$\begin{array}{rl} \text{P}(Z|{\text{Y}}_{m}^{c}) & =\text{P}(Z|{\text{X}}_{m}^{c},{\text{Y}}_{m}^{c})\text{P}({\text{X}}_{m}^{c}|{\text{Y}}_{m}^{c})+m\text{P}(Z|X_{1},{\text{X}}_{m}^{-c},{\text{Y}}_{m}^{c})\text{P}(X_{1},{\text{X}}_{m}^{-c}|{\text{Y}}_{m}^{c})\\ & =\text{P}(Z|{\text{X}}_{m}^{c})\text{P}({\text{X}}_{m}^{c}|{\text{Y}}_{m}^{c})+m\text{P}(Z|X_{1},{\text{X}}_{m}^{-c})\text{P}(X_{1},{\text{X}}_{m}^{-c}|{\text{Y}}_{m}^{c})\\ & =\frac{(1-p)\pi }{1-p\pi }\frac{\text{P}({\text{Y}}_{m}^{c}|{\text{X}}_{m}^{c})\text{P}({\text{X}}_{m}^{c})}{\text{P}({\text{Y}}_{m}^{c})}+m\frac{\text{P}({\text{Y}}_{m}^{c}|X_{1},{\text{X}}_{m}^{-c})\text{P}(X_{1},{\text{X}}_{m}^{-c})}{\text{P}({\text{Y}}_{m}^{c})}\\ & =\frac{1}{\text{P}({\text{Y}}_{m}^{c})}\left\{\frac{(1-p)\pi }{1-p\pi }{\left(1-q\right)}^{m}\text{P}({\text{X}}_{m}^{c})+mr{\left(1-q\right)}^{m-1}\text{P}(X_{1},{\text{X}}_{m}^{-c})\right\}\\ & =\frac{1}{\text{P}({\text{Y}}_{m}^{c})}\{(1-p)\pi {\left(1-q\right)}^{m}+pr{\left(1-q\right)}^{m-1}\pi \}\\ & =\frac{1}{\text{P}({\text{Y}}_{m}^{c})}{\left(1-q\right)}^{m-1}\{(1-q)(1-p)+pr\}\pi , \end{array}$$

where, using an argument similar to that used in the derivation of equation (3.1), we have used

$$\text{P}({\text{X}}_{m}^{c})=1-p\pi$$

and

$$\text{P}(X_{1},{\text{X}}_{m}^{-c})=\frac{\pi }{n}.$$

Similarly,

A 2 $$\begin{array}{rl} \text{P}(Z^{c}|{\text{Y}}_{m}^{c}) & =\text{P}(Z^{c}|{\text{X}}_{m}^{c})\text{P}({\text{X}}_{m}^{c}|{\text{Y}}_{m}^{c})+m\text{P}(Z|X_{1},{\text{X}}_{m}^{-c})\text{P}(X_{1},{\text{X}}_{m}^{-c}|{\text{Y}}_{m}^{c})\\ & =\left(1-\frac{(1-p)\pi }{1-p\pi }\right)\frac{\text{P}({\text{Y}}_{m}^{c}|{\text{X}}_{m}^{c})\text{P}({\text{X}}_{m}^{c})}{\text{P}({\text{Y}}_{m}^{c})}+m\cdot 0\cdot \text{P}(X_{1},{\text{X}}_{m}^{-c}|{\text{Y}}_{m}^{c})\\ & =\frac{1}{\text{P}({\text{Y}}_{m}^{c})}\left(1-\frac{(1-p)\pi }{1-p\pi }\right){\left(1-q\right)}^{m}(1-p\pi )\\ & =\frac{1}{\text{P}({\text{Y}}_{m}^{c})}{\left(1-q\right)}^{m}(1-\pi ). \end{array}$$

$\text{P}({\text{Y}}_{m}^{c})$ can be either derived directly or found by adding the numerators of equations (.1) and (.2), giving

$$\text{P}({\text{Y}}_{m}^{c})={\left(1-q\right)}^{m-1}\{(1-q)-(1-q-r)p\pi \}.$$

Equations (4.5) and (4.6) follow.
